# Application of Enhanced Recovery after Surgical Treatment of the Occipitocervical Region

**DOI:** 10.1111/os.13018

**Published:** 2021-05-05

**Authors:** Peng Liu, Hai Nie, Zhuan Wang, Bao Yao, Jia‐hong Li, Ji Zhou

**Affiliations:** ^1^ Department of Orthopaedic Surgery Eastern Hospital, Sichuan Academy of Medical Sciences & Sichuan Provincial People's Hospital Chengdu China

**Keywords:** Atlanto‐occipital joint, Enhanced recovery after surgery, Neck pain, Postoperative complications

## Abstract

**Objective:**

The concept of enhanced recovery after surgery (ERAS) has been proposed to provide guidance for the improved postoperative rehabilitation of patients with occipitocervical region disease (ORD).

**Methods:**

This study retrospectively investigated 208 consecutive patients (116 men and 92 women) ranging in age from 22 to 76 years with ORD between July 2014 and June 2017 in our medical center, who were divided into three groups that received different preoperative, intraoperative, and postoperative management plans: traditional group (*n* = 73), ameliorated group (*n* = 70), and ERAS group (*n* = 65). We compiled a range of data relating to demographics and postoperative changes in hemoglobin and albumin, surgery duration, intraoperative blood loss, number of postoperative hospitalization days and expenses, readmission rates, and visual analog scale pain symptoms. Data were statistically evaluated using one‐way analysis of variance with Student–Newman–Keuls‐*q* post hoc tests or chi‐square tests.

**Results:**

There were no significant differences in terms of age (*P* = 0.235), gender (*P* = 0.691), body mass index (*P* = 0.723), American Society of Anesthesiologists grade (0.747), lesion character (*P* = 0.337) and lesion site (*P* = 0.957) between the three groups. Within a 6 months follow‐up period, there was no significant difference between the three groups in terms of surgery duration (*P* = 0.225), blood loss (*P* = 0.172), changes in hemoglobin (*P* = 0.255) and albumin (*P* = 0.178). However, postoperative hospitalization days (*P* = 0.000), postoperative costs (*P* = 0.019) and improvement of pain symptoms (*P* = 0.000) in ERAS group were significantly lower or higher than those in traditional group or ameliorated group, respectively. There were 29 (39.73%), 22 (31.43%), and 13 (20.00%), recorded cases of postoperative complications in traditional group, ameliorated group and ERAS group, respectively; complications in ERAS group were significantly lower than those in other two groups (*P* = 0.043). Moreover, all of the complications were mitigated effectively by the infusion of fluid, analgesia, treatment of infections, or antiemetic medications. There were 2 (2.74%), 3 (4.29%) and 2 (3.08%), recorded cases of re‐admission in traditional group, ameliorated group and ERAS group, respectively, but there were no statistically significant differences when compared across the three groups (*P* = 0.866).

**Conclusions:**

ERAS can provide benefits when it applied to patients undergoing ORD surgery mainly in terms of reducing postoperative complications, however, ERAS does not increase the economic burden of patients or decrease the risk of readmission.

## Graphical abstract

This study investigated 208 consecutive patients to provide guidance for the improved postoperative rehabilitation of patients with occipitocervical region disease. We found enhanced recovery after surgery can provide benefits when it applied to patients undergoing occipitocervical surgery mainly in terms of reducing postoperative complications.

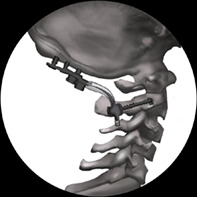

## Introduction

With recent developments in medical technology, the number of patients undergoing surgery of the craniocervical junction region is increasing year‐by‐year. This has increased the rate of treatment success for a variety of conditions, including atlantoaxial fractures, occipitocervical deformities, immune diseases, and subtentorial tumors^1^. Nevertheless, complications and unfortunate sequalae are not uncommon after such surgeries^2^, ^3^, and can lead to significant pain and suffering for patients and their family members.

In recent years, the concept of enhanced recovery after surgery (ERAS) has offered the potential to address these postoperative problems in many fields of surgery through the reduction of traumatic stress and the incidence of complications^4^, ^5^, ^6^, ^7^, as well as by promoting postoperative recovery and shortening hospital stays via the systematic application of multidisciplinary techniques during the perioperative period. These changes include the modification of anesthesia methods, surgical techniques, pain control protocols, blood management, and the adjustment of nursing care^8^. However, the application and effects of ERAS in occipitocervical junction surgery have not been adequately investigated.

This study retrospectively analyzed data from 208 patients who had undergone occipitocervical junction surgery, with or without ERAS. The clinical variables studied included blood loss, postoperative complications, nutritional status, hospital stay, and hospitalization costs. These variables were compared among different groups in order to: (i) provide better guidance for the postoperative rehabilitation of such patients; (ii) propose further discussions on the future of ERAS protocols for occipitocervical junction diseases; and (iii) optimize and increase the widespread implementation of ERAS protocols.

## Methods and Patients

### General Information

This retrospective study evaluated 301 patients with occipitocervical junction diseases who were treated between July 2014 and June 2017 in the orthopedic department and neurosurgery department of the Sichuan Provincial People's Hospital. Informed consent was provided by the patients prior to surgery. We also obtained approval from the institutional review board of our hospital to carry out this research. The study subjects consisted of 116 men and 92 women (age range: 22 to 76 years) who met the inclusion and exclusion criterion. Their diagnoses included lesions of the occipital bone (*n* = 55), including occipital meningiomas (*n* = 28), tumors of the cerebellar vermis (*n* = 10), cerebellar hemorrhages (*n* = 13), and Chiari malformations (*n* = 4). The lesions were located in the occipital bone and atlas (*n* = 88), including Klippel–Feil malformations (*n* = 12), bone fractures and/or dislocation (*n* = 38), single basilar invaginations (*n* = 13), basilar invaginations complicated by atlantoaxial instability (*n*=18), and basilar invaginations complicated by platybasia (*n* = 7). The pathology was centered in the atlas and/or axis (*n* = 65), including odontoid process fractures (*n* = 31), atlantoaxial dislocations (*n* = 12), atlantoaxial instability (*n* = 8), and odontoid deformities (*n* = 14).

### Inclusion and Exclusion Criteria

Inclusion criteria: (i) atlantoaxial fracture and dislocation; (ii) occipitocervical deformity; (iii) single rheumatoid lesions of the occipitocervical junction; and (iv) subtentorial tumors.

Exclusion criteria: (i) patients with complete neurological damage; (ii) fracture and/or dislocation of the upper cervical spine with poly trauma; (iii) surgery without opening spinal canal; (iv) malignant tumors and infectious diseases; (v) complications of diabetes mellitus, or more than two systematic medical diseases; (vi) a history of blood or albumin (ALB) transfusion: and (vii) incomplete hospitalization data.

### Indications for Surgical Treatment

Surgical indications included severe pain, neurological deficits, and instability of the spine, as well as lesions in the posterior cranial fossa, meningiomas without calcification but more than 2 cm in diameter, cerebellar hemorrhage over 10 ml in volume, and Arnold–Chiari malformation with progressive dyspnea, syringomyelia, and deformities of the foramen magnum.

### Preoperative Diagnosis

The local availability of X‐ray, computed tomography (CT), magnetic resonance imaging, and CT angiography was necessary. There were two common manifestations of these diseases in imaging findings: (i) the imaging findings of spinal instability included change of radian, a step‐like connecting line at the posterior edge of the vertebral body, bilateral sign of posterior margin of vertebral body and the double features of the facet joints of the cervical spine; and (ii) the abnormal mesenchymal or nerve tissues around the lesions in magnetic resonance imaging examinations included high signals from brain tissues, spinal cord, ligaments, or muscles under the T2 WI.

### Surgery and interventions

#### Anesthesia and Position

Skull traction bows were used after anesthesia, and the patients were placed in the prone position on the operating room table. Then, the patients were fixed in an appropriate position such that the shoulders were parallel, and the cervical spine was without deflection or rotation.

#### Approach and Surgical Procedure

In the early stages of surgery, the occipital bone and upper cervical vertebra were exposed through a posterior median approach. The subsequent procedures were determined by the following types of lesions: (i) patients with upper cervical trauma had pedicle or occipital screws placed under navigation, and their subsequent procedures included reduction, fixation, and the placement of bone grafts; (ii) for patients with occipitocervical malformations, both resection of the posterior arch of the atlas, and the enlargement of the foramen magnum with a high‐speed drill, were necessary for nerve decompression. As for those with Chari malformation, the fourth ventricle central aqueduct and the superior cervical medulla were explored after the hernia had been removed under microscopy. Then, screws were placed, and reduction and fixation were executed, supplemented with large bone grafts from the iliac crest; and (iii) for occipital lesions, the occipital bone was trimmed using a rongeur, the lesions were resected, and the dura was repaired.

#### Group Allocations

According to different preoperative, intraoperative, and postoperative interventions including preoperative preparation, measures to reduce surgical injury, rehabilitation training and nutrition support, three groups of patients were included in this study: traditional group (*n* = 73), ameliorated group (*n* = 70), and ERAS group (*n* = 65) (Table 1).

**TABLE 1:** Patient management methods during perioperative period

Groups	Preoperative	Intraoperative	Postoperative
Traditional group (*n* = 73)	Traditional preoperative preparation	Traditional surgery	Prevention of thrombosis and pneumonia Functional training
Ameliorated group (*n* = 70)	Traditional preoperative preparation	Control of body temperature and blood pressure Navigation and minimal invasive surgery*** Bone tissue engineering**** Dura patch***** Electrophysiological monitoring Local anesthesia and hemostatic drugs******	Prevention of thrombosis and pneumonia Functional training
ERAS group (*n* = 65)	Strengthen preoperative education Shorten fasting time* Preemptive analgesia**	Control of body temperature and blood pressure Navigation and minimal invasive surgery*** Bone tissue engineering**** Dura patch***** Electrophysiological monitoring Local anesthesia and hemostatic drugs******	Prevention of thrombosis, pneumonia, nausea and vomiting Functional training Ladder for analgesia Assisted sedation Promoting sleep Promoting hematopoiesis and nutritional support

*Preoperative fasting time was shortened to 2 hours through use of feeding nutrient solution

**Oral celecoxib was administered 2 days before surgery

***Application in cases in which pedicle screws were used

****Application in cases of bone grafts

*****Application in cases of operation involving the spinal canal, or within the skull

******tranexamic acid and ropivacaine were utilized in the wound cavity and subcutaneously.

### Extraction of Medical History Data

#### Demographic Data and Anesthesia Evaluation of Patients

We scrutinized each patient's medical chart and recorded selected demographic data including age, gender, body mass index and American Society of Anesthesiologists grade to ensure the homogeneity of patients' condition (Table 2).

**TABLE 2:** Demographic data for occipitocervical region disease (ORD) patients

Groups	Years	Gender (cases)		BMI (kg/m^2^)	ASA grade (cases)		
		Male	Female		I	II	III
Traditional group (*n* = 73)	40.67 ± 11.43	40	33	22.24 ± 3.19	40	29	4
Ameliorated group (*n* = 70)	42.53 ± 11.84	37	33	22.42 ± 4.25	34	30	6
ERAS group (*n* = 65)	39.29 ± 9.72	39	26	21.94 ± 2.70	37	22	6
χ^2^/*F*	1.457	0.740		0.325	1.939		
*P*	0.235	0.691		0.723	0.747		

#### Surgical Indicators and Postoperative Nutritional Status of patients

The duration of the surgical procedure and intraoperative blood loss were used to assess surgery performance. The change of postoperative hemoglobin and ALB were recorded to detect patients' nutritional status.

#### Perioperative Complications

Adverse consequences that occurred during the surgical treatment of these diseases were recorded to evaluate the surgery safety.

#### Burden of Patients

The number of days hospitalization postoperatively and postoperative expenses were collected to investigate patients' burden.

#### Follow Up

Visual analogue scale was adopted to assess the surgical site pain within six months after surgery. Out of a total score of 10, 0 corresponds to no pain, 1–3 points to mild pain, 4–6 points to moderate pain, 7–9 points to severe pain, and 10 refers to unbearable pain. Readmission episodes were collected to evaluate curative effect.

### Statistical Analyses

Data were expressed as mean ± standard deviation. The differences in means between groups were statistically evaluated by one‐way analysis of variance with the Student–Newman–Keuls‐*q* post hoc test or chi‐square tests. All analyses were performed using Statistical Product and Service Solutions software, version 16.0 (SPSS UK, Ltd., Woking, UK) and *P* < 0.05 was considered to be statistically significant.

## Results

### Patient General Data

Mean patient age in the traditional group, ameliorated group and ERAS group was 40.67 ± 11.43, 42.53 ± 11.84, and 39.29 ± 9.72 years, respectively; there were no statistically significant differences between the three groups (*P* = 0.235). There were no significant gender differences between the three groups (*P* = 0.691). Moreover, there were no significant differences between the three groups in terms of American Society of Anesthesiologists grade (*P* = 0.747). Furthermore, there were no significant differences in terms of lesion character, and lesion site between the three groups (Table 3).

**TABLE 3:** The characteristic and site of lesion

Groups	Characteristic of lesion						Site of lesion		
	Fracture	Dislocation	Tumor	Hemorrhages	Deformity	Instability	Occipital bone	Occipital bone and atlas	Atlas and/or axis
Traditional group (*n* = 73)	19 (26.03%)	3 (4.11%)	20 (27.40%)	4 (5.48%)	24 (32.88%)	3 (4.11%)	20 (27.40%)	31 (42.47%)	22 (30.14%)
Ameliorated group (*n* = 70)	29 (41.43%)	3 (4.29%)	8 (11.43%)	6 (8.57%)	22 (31.43%)	2 (2.86%)	20 (28.57%)	29 (41.43%)	21 (30.00%)
ERAS group (*n* = 65)	21 (32.31%)	6 (9.23%)	10 (15.38%)	3 (4.62%)	22 (33.85%)	3 (4.62%)	15 (23.08%)	28 (43.08%)	22 (33.85%)
χ^2^	11.264						0.650		
*P*	0.337						0.957		

### Surgical and Postoperative Conditions of Patients

The key point of surgery is to ensure careful operation in this area to avoid nerve injury. The mean surgery time in traditional group, ameliorated group and ERAS group was 179.95 ± 45.37, 184.62 ± 66.08, and 169.28 ± 42.26 min, respectively; there was no statistically significant difference between the three groups (*P* = 0.225). The mean blood loss during surgery in the traditional group, ameliorated group and ERAS group was 1004.20 ± 217.91, 940.51 ± 246.84, and 939.42 ± 239.32 mL, respectively; there was no statistically significant difference between the three groups (*P* = 0.172). The mean hemoglobin reductions in the patients one week after surgery in traditional group, ameliorated group and ERAS group was 23.25 ± 10.83, 21.02 ± 10.13, and 20.20 ± 12.74 g/L, respectively (*P* = 0.255). The mean ALB reductions in the three groups one week after surgery was 9.41 ± 4.81, 8.03 ± 4.95, and 8.46 ± 3.63 g/L, in traditional group, ameliorated group and ERAS group, respectively (*P* = 0.178); there were no statistically significant differences between the three groups.

### Adverse Events

There were 29 (39.73%), 22 (31.43%), and 13 (20.00%), recorded cases of postoperative complications in the traditional group, ameliorated group, and ERAS group, respectively. Moreover, the ERAS group decreased by 55.17% and 40.91% lower than the other two groups, these differences were significant (*P* = 0.043). The complications mostly involved the leakage of cerebrospinal fluid, incision complications (edema, ecchymosis, hematoma, and infection), organ infections (mainly were pneumonia and urinary tract infection), and nausea or vomiting (Table 4). Moreover, all of the complications were mitigated effectively by the infusion of fluid, analgesia, treatment of infections, or antiemetic medications. There were four recorded cases of other concomitant complications in traditional group (5.48%). Extrapyramidal symptoms occurred in two cases, and hemiplegia, hemianopia, aphasia, and atelectasis, occurred in one case each. All of these conditions resolved with time. Similarly, there were five cases of concomitant complications in ameliorated group (7.14%). One case, involving extrapyramidal deficits, which gradually improved after neurotrophic therapy. In one case, a small intracranial hematoma was fully absorbed in 4 weeks. One case of hyperhidrosis healed after 2 months without specific treatment. One subject suffered from generalized pain, and one other case experienced dyspnea; both were cured by symptomatic treatment within one week. In the ERAS group, there were four cases of concomitant complications (6.15%). Atelectasis and ileus occurred in two patients, although both symptoms disappeared after specific treatment. One patient experienced facioplegia, although this was relieved by neurotrophic therapy and acupuncture. Quadriplegia appeared in one patient but resolved spontaneously within 2 weeks.

**TABLE 4:** Surgery‐relevant and hospitalization data for occipitocervical region disease (ORD) patients

	Variable	Traditional group (*n* = 73)	Ameliorated group (*n* = 70)	ERAS group (*n* = 65)	χ^2^/*F*	*P*
Surgery and hospitalization (mean ± SD)	Surgery time(min)	179.95 ± 45.37	184.62 ± 66.08	169.28 ± 42.26	1.503	0.225
	Blood loss (mL)	1004.20 ± 217.91	940.51 ± 246.84	939.42 ± 239.32	1.774	0.172
	Postoperative hospitalization (day)	17.39 ± 3.37	16.20 ± 3.56	14.36 ± 2.57	15.41	0.000 ^***^
	Postoperative cost (RMB)	19387.60 ± 4076.32	18017.33 ± 3288.02	17962.48 ± 2525.35	4.035	0.019^*^
Change after surgery (mean ± SD)	Hemoglobin (g/L)	23.25 ± 10.83	21.02 ± 10.13	20.20 ± 12.74	1.376	0.255
	Albumin (g/L)	9.41 ± 4.81	8.03 ± 4.95	8.46 ± 3.63	1.740	0.178
	Improvement of VAS score	2.34 ± 1.47	2.57 ± 1.21	3.54 ± 1.54	13.603	0.000^***^
Perioperative complications	Cerebrospinal fluid leakage	6 (8.22%)	5 (7.14%)	2 (3.08%)	6.302	0.043 ^*^
	Wound complication	7 (9.59%)	3 (4.29%)	2 (3.08%)		
	Another organ infection	5 (6.85%)	5 (7.14)	4 (6.15%)		
	Nausea and vomiting	11 (15.07%)	9 (12.86)	5 (7.69%)		
Readmission	Rate	2 (2.74%)	3 (4.29%)	2 (3.08%)	0.287	0.866

*p < 0.05, ***p < 0.001

### Postoperative Hospitalization and Expenses

Furthermore, the mean duration of postoperative hospitalization in the traditional group, ameliorated group and ERAS group, was 17.39 ± 3.37, 16.20 ± 3.56, and 14.36 ± 2.57 days, respectively (*P* = 0.000) while the mean postoperative costs in the traditional group, ameliorated group and ERAS group, were 19387.60 ± 4076.32, 18017.33 ± 3288.02, and 17962.48 ± 2525.35 RMB, respectively (*P* = 0.019); the ERAS group decreased by 17.42% and 11.36%, 7.35% and 0.30% lower than other two groups in terms of days and costs, respectively. These differences were statistically significant.

### Improvement of Pain Symptoms and re‐admission

The improvement of visual analog scale in the traditional group, ameliorated group and ERAS group, was 2.34 ± 1.47, 2.57 ± 1.21, 3.54 ± 1.54, respectively (*P* = 0.000); the ERAS group increased by 51.28% and 37.74% more than other two groups, the difference was statistically significant. There were two (2.74%), three (4.29%) and two (3.08%), recorded cases of re‐admission in the traditional group, ameliorated group and ERAS group, respectively, but there were no statistically significant differences when compared across the three groups (*P* = 0.866).

## Discussion

### Efficacy of ERAS and Its Principle

#### Relief of Pain and Anxiety

The results of the present study indicate that there is a clear superiority of use of ERAS for patients undergoing spinal surgery and neurosurgery, as evidenced by the shortening of hospital stays, along with the reduction of post‐operative pain and complications. However, there are many factors that might be responsible for these improvements. Preemptive analgesia, with oral celecoxib, is also known to play an important role in alleviating post‐operative pain^9^, ^10^. A recent systematic review showed that although there is no clear evidence that preoperative education can reduce postoperative pain and complications or the duration of hospitalization, it can significantly alleviate anxiety^11^. Compared with other types of surgery, patients have obviously higher levels of anxiety and fear with regards to operations involving the head and neck. This is an important factor to consider, as anxiety and fear can aggravate stress responses after surgery^12^. Therefore, the medical staff who interact with these patients need to have good communication skills, so that patients can fully understand their disease conditions and corresponding treatment methods. Although this would help to reduce unnecessary fear and unfavorable stressors, and thus enable each patient to better cooperate with the treatment, this has rarely been reported in previous studies.

#### Alleviation of Inflammation

Adverse postoperative reactions are mainly associated with the stress response following surgery. This surgical stress response can be divided into two types: inflammation arising from an imbalance between pro‐inflammatory and anti‐inflammatory cytokines, and the catabolism and increased cardiovascular metabolism that occurs in response to systemic metabolic requirements^13^. The application of ERAS leads to improved surgical technology and better perioperative management, thus reducing the stress response to surgery; this leads to a consequential reduction in postoperative complications^13^, ^14^.

#### Nutrition and Postoperative Rehabilitation Training

Controlling body temperature and blood pressure can reduce postoperative blood loss and maintain the balance of hydration and electrolytes, which is conducive to reducing body consumption and maintaining normal organ function; this reduces hematoma and wound infiltration with blood, thus avoiding delayed wound healing. We think that shorter fasting times and preoperative preemptive analgesia may reduce the risks of wound complications such as edema, ecchymosis, hematoma, and infection, as may postoperatively promoting hematopoiesis and nutritional support of ERAS. Shorter preoperative fasting times and utilizing postoperative nutritional support can also reduce body consumption and increase the patient's energy, which is conducive to the repair of damaged tissues, enhances immunity, and reduces the risk of infection. Tarrant *et al*.^15^ reported that an abnormally low preoperative body mass index was significantly associated with postoperative wound infection and weight loss. The children's spinal surgery guidelines^16^ also state that preoperative nutritional evaluation and perioperative nutritional support are conducive to the prevention of wound infection. Analgesia has a positive effect on the prevention of wound complications, and it is conducive to functional exercise. Under the protection of a neck circle, the occipital and cervical muscles contract to similar lengths, which promotes blood circulation within local soft tissues. This accelerates the regression of swelling and hematoma absorption, and reduces inflammation. Conversely, pain can exacerbate anxiety^17^, and patients are more likely to remain bedridden for a longer period in a negative state and compress the wound, which delays wound healing and can promote infection.

#### Infection Prevention

In our study pertaining to postoperative organ infections are mainly focused on pneumonia and urinary tract infection. Most infections were pulmonary, and these patients were divided into two main categories. One category was infections caused by the lung itself, including pneumonia and atelectasis. These patients commonly exhibited poor lung function or pain and anxiety originating from surgery. Preemptive preoperative analgesia and postoperative nutritional support of ERAS effectively reduce these complications. The other category was infections caused by poor respiratory function resulting from nerve injury. In this regard we mainly discuss the injury involved during and after surgery. Intraoperative injury is usually caused by clearing hematoma, peeling off a tumor, reduction, and screw insertion, and such injuries can be reduced via navigational and electrophysiological detection aids. Postoperative injury is mainly caused by hematoma and ischemia–reperfusion, but minimally invasive technology can reduce its occurrence. Urinary tract infection often occurs in patients who have been bedridden for a long time and/or have had an indwelling catheter for a long time. Such patients are typically not in good general condition. Minimizing the likelihood of such problems requires adherence to every aspect of ERAS, which can promote early extubation and functional exercise, thus reducing their incidence.

#### Recent Developments of REAS

Over recent years, there has been significant development in new surgical techniques, including 3D navigation to help localize the sites that require surgical intervention. In particular, during upper cervical procedures, the safety of the instrumentation step has been increased markedly via the use of surgical navigation^18^. During occipital surgery, deep brain lesions, or tumors with unclear margins, can be approached more precisely using these newer navigation systems^19^. Thus, these new techniques can improve accuracy, reduce radiation exposure, shorten the duration of surgery, and increase patient safety^20^. These novel navigation techniques embody precision medicine and facilitate the use of minimally invasive procedures, thus reducing tissue damage and inflammation, and consequentially reducing the incidence of post‐operative complications^21^. In addition, other newer technologies, such as 3D printing and robotic surgery, have led to significant advancement in the precision of surgery performed in the occipitocervical region. These developments should markedly improve the success rate of such procedures, reduce the need for reoperations, and thus shorten the meantime‐to‐discharge, as well as reducing readmission rates^22^, ^23^.

The application of intraoperative electrophysiological monitoring has effectively reduced the incidence of nerve injuries, including somatosensory evoked potentials, motor evoked potentials, and electromyography. This monitoring procedure has also improved the success rate of surgery and reduced re‐operative rates, length‐of‐stay as an inpatient, and readmission rates^24^. The results of these previous studies are consistent with our present findings^25^, ^26^. Another technique that we used intraoperatively was bone tissue engineering; this involved a piece of osteogenic periosteum being placed on the surface of the graft. The periosteum contains osteo inductive factors that exert pro‐osteogenic and anti‐osteoclastic properties; it is clear that these implants have the potential to improve bone regeneration in the spine by promoting bone fusion^27^. Thus, in theory, the development of this new technology could lead to reduced levels of postoperative pain (arising from non‐union) and reduce readmission rates.

#### Application of REAS in Occipitocervical Surgery

In the early stages of recovery from spinal surgery, there is usually intense pain. Previous studies have shown that the several most painful surgeries involve vertebral fusion and complex spinal reconstruction^28^. In addition to incisional pain, severe pain can also be caused in these patients by deep tissue trauma to the spinal ligaments, muscles and periosteum. Moreover, the presence of the cervical plexus, and abundant peripheral nerves, around the spinal facet joints can also cause deep body pain and severe reflex spasmodic pain in adjacent spinal cord segments^29^. It has become very evident that incomplete pain control can lead to serial adverse events. Therefore, adequate local anesthesia should be a conventional approach to surgical incisions involving the cervical spine. The use of local anesthetics in this setting not only reduces the stress response, but also reduces the need for intravenous analgesics, thereby reducing postoperative nausea and vomiting.

Compared to other forms of surgery, the leakage of cerebrospinal fluid is a major factor affecting postoperative wound healing in the occipitocervical region, especially in neurosurgery, where the dura mater must be opened^30^, ^31^. The traditional management of this complication includes the placement of wound sutures, the application of pressure bandages, bed rest, and adjustments to the height of the patient's head. If these interventions are ineffective, continuous lumbar drainage should be performed, and reoperation should be considered^32^. Over recent years, some biomaterials have shown good results in laboratory conditions^33^, ^34^; these also proved effective in our present study. In the ERAS group, the mere application of a patch of artificial dura material significantly reduced the incidence of cerebrospinal fluid leakage. Another reason for poor wound healing is the local anatomy. This region is susceptible to pressure, and has abundant sweat glands and sebaceous glands. Therefore, infections of the incision site are common complications. Reducing the postoperative duration in the supine position, as well as providing good wound dressings, room temperature control, and appropriate antibiotic treatment, should be considered under all circumstances.

#### Widespread Implementation of ERAS

The optimization and popularization of ERAS requires the cooperation of medical staff, patients, and society. The first step should be establishing a leading ERAS group in the hospital, composed of multidisciplinary teams including the leaders of disciplines such as surgery, anesthesia, nursing, nutrition, rehabilitation, and psychology^35^, ^36^. In accordance with some published guidelines consensus and operation specifications, the core ERAS measures and process specifications that meet the required standards and are scientific, reasonable, and suitable for both doctors and patients should then be established and strictly implemented. They should be diligently monitored thereafter, in an effort to identify areas of potential improvement. It is also important that the benefits associated with optimized rehabilitation are emphasized to doctors, especially young residents. All staff involved in patient care should be aware that different patients have different levels of awareness and consequent participation. Thus, it is necessary to implement individualized therapeutic schedules and comprehensive health education based on patients' individual circumstances, in an effort to improve patient participation. Lastly, the widespread implementation of ERAS requires the strong support of hospital management departments and the government, via measures such as opening relevant green channels and implementing relevant medical insurance policies.

#### Limitations

Although the present study covered a variety of diseases and a wide ranging in age, their distribution among groups was balanced. This ensures homogeneity and comparability between groups. Of course, some limitations that need to be taken into consideration. For instance, it is unclear whether the use of ERAS increased the risk of spinal non‐fusion owing to the short follow‐up time.

### Conclusions

Based on multimodal methodology, ERAS can provide additional benefits to patients after occipitocervical surgery. These benefits mainly result from the reduction of postoperative complications. However, ERAS does not increase the economic burden of patients or decrease the risk of readmission.

### Ethical Approval and Informed Consent

The study protocol was approved by our Institutional Review Board (IRB) at Sichuan Provincial People's Hospital, Sichuan Academy of Medical Science, Chengdu, People's Republic of China. Written and informed consent was obtained from all patients for the publication of this study.
